# Draft Genome Sequence of a Rhodococcus Strain Isolated from Tannery Wastewater Treatment Sludge

**DOI:** 10.1128/genomeA.01463-14

**Published:** 2015-01-29

**Authors:** Ji-Quan Sun, Lian Xu, Li-Juan Wang, Xiao-Lei Wu

**Affiliations:** a College of Engineering, Peking University, Beijing, China; b Institute of Engineering (Baotou), College of Engineering, Peking University, Baotou, Inner Mongolia, China

## Abstract

Rhodococcus sp. Chr-9 can degrade pyridine in the presence of chromate. Its draft genome sequence revealed that strain Chr-9 harbors sets of genes for resistance to heavy metals such as lead, mercury, arsenate, and cobalt, as well as three different gene clusters for metabolizing aromatic compounds, such as phenol, benzoate, and 4-nitrophenol.

## GENOME ANNOUNCEMENT

Heavy metals and aromatic compounds are often found in the environment simultaneously, resulting in aromatic-heavy metal cocontamination, which makes the biodegradation of aromatics more difficult (1). Gram-positive strain Chr-9, belonging to Rhodococcus according to the 16S rRNA gene as well as its morphological and physiological characteristics, was isolated from the sludge of a tannery wastewater reactor in Binzhou, Shandong Province, China. It was capable of degrading phenol and pyridine effectively in the presence of hexavalent chromium and of removing hexavalent chromium in a rich medium (1, 2). Therefore, we sequenced its draft genome.

Rhodococcus sp. Chr-9 was cultured in Luria-Bertani medium (10 g·liter^−1^ NaCl; 5.0 g·liter^−1^ yeast extract; 10 g·liter^−1^ tryptone, pH 7.0) and genomic DNA was prepared using a DNA extraction kit (BioTech) following the manufacturer’s instruction. The genome was sequenced using Illumina paired-end technology (3). Briefly, genomic libraries containing 500-bp paired-end sequences were constructed and sequenced with Illumina Genome Analyzer IIx, giving 100-fold coverage of the genome. Consequently, 3,077,861 paired-end reads were generated and *de novo* assembled using Velvet 1/2.09 (4), generating 144 contigs longer than 200 bp with a maximum length of 834 kbp. The total length of the contigs assembly was 5.35 Mbp, and the G+C content was 67.7%. A total of 5,050 candidate protein coding genes as well as 48 tRNAs and only 1 rRNA operon, were assigned by automated annotation of the Rhodococcus sp. Chr-9 draft genome sequence using Prodigal (5) and Rapid Annotation using Subsystem Technology (RAST) server version 4.0 (6). Comparative genome analysis performed on the Rhodococcus pyridinivorans stain SB3094 genome (GenBank accession no. NC_023150.1) using MUMmer (7) revealed that 97% of SB3094 can be aligned with Chr-9 with an average of 90% identity.

In the genome of Rhodococcus sp. Chr-9, two clusters involved in phenolic compounds were found. The first is cluster *pheA_1_A_2_* responsible for phenolic compound 2-hydroxylation coupled with *catRABC* catalyzing the phenolic ring indiol-cleavage and the following reactions. The second is cluster *pbhA_1_A_2_BCDE* responsible for 4-nitrophenol degradation, *pbhA_1_A_2_* responsible for 4-nitrophenol 2-hydroxylation, while *pbhBCDE* catalyzes the phenolic ring extra-cleavage, and the following reactions. Furthermore, a cluster *benABCDKE* involved in benzoate transportation and degradation, and two cytochrome P450 genes catalyzing phenolic compound hydroxylation were also detected. As for heavy metal resistance genes, arsenic resistance operons (*arsADC_1_RBC_2_C_3_C_4_C_5_*) and eight scattered arsenate reductase genes, mercuric ion reductase gene *merA* and alkylmercury lysase *merB*, and eleven lead-cadmium-zinc-mercury transporting genes were detected in the genome. Furthermore, two sets of cobalt-zinc-cadmium resistance genes *cszD*, two sets of cadmium resistance transporter genes *cadD*, two sets of TauX superfamily nikel/cobalt transporter genes, magnesium and cobalt transport gene *corA*, and copper resistance genes *copC* and *copD* were also found. However, no hexavalent chromium reductase, reported earlier, was found in the genome.

### Nucleotide sequence accession numbers.

The whole-genome shotgun project of Rhodococcus sp. Chr-9 has been deposited at GenBank under the accession no. JTIZ00000000. The version described in this paper is the first version, number JTIZ01000000.
